# Comparison of sputum collection methods for tuberculosis diagnosis: a systematic review and pairwise and network meta-analysis

**DOI:** 10.1016/S2214-109X(17)30201-2

**Published:** 2017-06-15

**Authors:** Sumona Datta, Lena Shah, Robert H Gilman, Carlton A Evans

**Affiliations:** a Innovation For Health and Development, Laboratory of Research and Development, Universidad Peruana Cayetano Heredia, Lima, Peru; b Infectious Diseases and Immunity, Imperial College London and Wellcome Trust Imperial College Centre for Global Health Research, London, UK; c Innovacion Por la Salud Y el Desarrollo, Asociación Benéfica Prisma, Lima, Peru; d Department of Epidemiology, Biostatistics and Occupational Health, McGill University, Montreal, QC, Canada; e Department of International Health, Johns Hopkins Bloomberg School of Public Health, Baltimore, MD, USA

## Abstract

**Background:**

The performance of laboratory tests to diagnose pulmonary tuberculosis is dependent on the quality of the sputum sample tested. The relative merits of sputum collection methods to improve tuberculosis diagnosis are poorly characterised. We therefore aimed to investigate the effects of sputum collection methods on tuberculosis diagnosis.

**Methods:**

We did a systematic review and meta-analysis to investigate whether non-invasive sputum collection methods in people aged at least 12 years improve the diagnostic performance of laboratory testing for pulmonary tuberculosis. We searched PubMed, Google Scholar, ProQuest, Web of Science, CINAHL, and Embase up to April 14, 2017, to identify relevant experimental, case-control, or cohort studies. We analysed data by pairwise meta-analyses with a random-effects model and by network meta-analysis. All diagnostic performance data were calculated at the sputum-sample level, except where authors only reported data at the individual patient-level. Heterogeneity was assessed, with potential causes identified by logistic meta-regression.

**Findings:**

We identified 23 eligible studies published between 1959 and 2017, involving 8967 participants who provided 19 252 sputum samples. Brief, on-demand spot sputum collection was the main reference standard. Pooled sputum collection increased tuberculosis diagnosis by microscopy (odds ratio [OR] 1·6, 95% CI 1·3–1·9, p<0·0001) or culture (1·7, 1·2–2·4, p=0·01). Providing instructions to the patient before sputum collection, during observed collection, or together with physiotherapy assistance increased diagnostic performance by microscopy (OR 1·6, 95% CI 1·3–2·0, p<0·0001). Collecting early morning sputum did not significantly increase diagnostic performance of microscopy (OR 1·5, 95% CI 0·9–2·6, p=0·2) or culture (1·4, 0·9–2·4, p=0·2). Network meta-analysis confirmed these findings, and revealed that both pooled and instructed spot sputum collections were similarly effective techniques for increasing the diagnostic performance of microscopy.

**Interpretation:**

Tuberculosis diagnoses were substantially increased by either pooled collection or by providing instruction on how to produce a sputum sample taken at any time of the day. Both interventions had a similar effect to that reported for the introduction of new, expensive laboratory tests, and therefore warrant further exploration in the drive to end the global tuberculosis epidemic.

**Funding:**

Wellcome Trust, Joint Global Health Trials consortium, Innovation For Health and Development, and Bill & Melinda Gates Foundation.

## Introduction

Globally, tuberculosis affects around 10·4 million people per year, and kills around 1·4 million of them, with the majority of patients presenting with pulmonary disease.^1^ One of the key challenges to global tuberculosis control is correct diagnosis, and WHO has prioritised improving diagnostic guidelines and tests.^2^ However, the sensitivity of a diagnostic test depends on the quality of the sputum samples obtained,^3^, ^4^ which has been the subject of much less attention than the development of new diagnostic products.

Microscopy is inexpensive and the most frequently used laboratory test globally, but it is only likely to be positive if the concentration of acid-fast *Mycobacterium tuberculosis* bacilli exceeds 10 000 per mL of sputum.^5^ Tuberculosis culture techniques generally have greater sensitivity than microscopy, and PCR has intermediate sensitivity between that of culture and microscopy. However, culture and PCR tests, which are more expensive than microscopy, can only diagnose tuberculosis in samples containing sufficient concentrations of *M tuberculosis*. Thus, poor quality sputum samples can lead to missed tuberculosis diagnoses for all tests, since diagnostic sputum samples inevitably contain respiratory secretions from both the healthy airway tract and the diseased lung together with variable amounts of saliva. Consequently, the positivity of laboratory tests often varies between samples from the same patient,^6^ so more than one sputum sample is usually tested from each person with suspected tuberculosis.^7^, ^8^ Recommended sputum collection methods vary globally^9^, ^10^ and their relative merits for tuberculosis diagnosis are poorly characterised. Reviews have been done to determine the number of sputum samples required, and the number of days over which they should be collected.^7^, ^11^ However, we could not identify any published systematic review or meta-analysis assessing how best to collect sputum samples to improve tuberculosis diagnosis.

**Research in context**

**Evidence before this study**

We searched PubMed, Google Scholar, ProQuest, Web of Science, CINAHL, and Embase for studies published up to April 14, 2017, that assessed the effect of sputum collection methods on tuberculosis diagnosis in adults. Our search identified reviews on the number of serial sputum samples required, including the possibility of collecting these over the same day and reducing the risk of patient dropout. Both reviews have contributed to sputum collection guidelines. These guidelines also include diverse recommendations on sputum production and collection methods, but we found no published meta-analysis supporting these recommendations. A systematic review published in 2015 investigated the effect of two sputum collection methods on indirect markers of sputum quality, but cast doubt on the relevance of these indirect markers for tuberculosis diagnosis. Most research studied the use of sputum induction or bronchoscopy to improve sputum collection and consequently tuberculosis diagnosis. However, these invasive techniques have limited applicability in resource-constrained, community settings where the majority of the world's tuberculosis occurs.

**Added value of this study**

This is, to our knowledge, the first systematic review and meta-analysis studying the effect of non-invasive sputum collection methods on tuberculosis diagnosis. We found that sputum collection methods significantly affect diagnosis of tuberculosis. Either pooling sputum collection or providing instruction and assistance almost doubled the odds of correctly diagnosing a person with tuberculosis. We anticipate that implementation of these recommendations will be inexpensive and feasible for resource-constrained settings.

**Implications of all the available evidence**

Patient-centred care to diagnose tuberculosis early is a prime component of the WHO End TB Strategy. Widening this focus to include sputum collection interventions can improve diagnosis with the available tools. However, further research is required on this topic, especially concerning practical techniques to improve sputum production. Our findings have the potential to be rapidly implemented in the field since new diagnostic tests require substantial investment and time to be implemented, whereas improvement of sputum collection might have a similar effect on case finding but within a shorter timeframe. These findings could facilitate the WHO objective to end the tuberculosis epidemic by 2035.

A systematic review^12^ evaluated the effect of two pre-collection interventions (instructions and mouth washing) on indirect indicators of sputum quality, such as sputum viscosity. The heterogeneity and paucity of data provided no definitive conclusions and questioned the validity of using these indirect sputum quality indicators to predict accurate diagnosis.^12^ Other studies have assessed the utility of techniques that we categorised as invasive, such as sputum induction and bronchoscopy, but these techniques have limited applicability in the resource-constrained settings where most tuberculosis occurs, because of biosafety issues, equipment availability, and human capacity.^13^ We therefore aimed to do a systematic review and meta-analysis of the effects of non-invasive sputum collection methods on correct diagnosis in people aged at least 12 years and suspected of having pulmonary tuberculosis. We excluded children younger than 12 years because both expectoration of sputum and laboratory confirmation of tuberculosis are uncommon in this age group.^14^

## Methods

### Search strategy and selection criteria

This systematic review and meta-analysis was done according to a protocol based on international standards that was developed before data collection commenced.^15^, ^16^, ^17^ We searched PubMed, Google Scholar, ProQuest, Web of Science, CINAHL, and Embase for studies published up to April 14, 2017, using the following search terms: “tuberculosis” or “TB”; “sputum”; “collection” or “clearance” or “submission” or “acquisition”; “technique” or “guide” or “method”; and “diagnosis”. Additionally, references cited by these publications and relevant articles that we identified were hand-searched.

For inclusion in the study, full-text, peer-reviewed articles in English were selected that described experimental, case-control, or cohort studies comparing the effects of any two non-invasive sputum collection methods on the diagnostic performance of laboratory tests for pulmonary tuberculosis in people aged at least 12 years. Studies that also evaluated invasive collection methods were included, but we only considered the comparison of non-invasive sputum collection methods. Studies were included regardless of co-infection with HIV and publication date. We did not include studies that only assessed sputum quality or culture contamination rates, without assessing diagnostic performance.^12^ To ensure that findings of previous systematic reviews were not repeated, studies were excluded if they investigated only the effects of sputum storage, collection of samples on the same versus different days, or the incremental yield of second and third sputum samples.^7^, ^11^

SD and CAE applied the inclusion and exclusion criteria and grouped studies comparing the following aspects of sputum collection methods: sputum collection time and duration; and sputum collection instructions and techniques. SD and LS used a form (appendix pp 1–6) to extract the data in duplicate. The following data were extracted from each study: research question, number of sputum samples, sputum collection characteristics, exact instructions given and method, sample transport details, sputum processing, laboratory methods, and sample volume. Laboratory test results were extracted for diagnostic performance (positive results as a proportion of all positive and negative results) and for microscopy also the concentration of acid-fast bacilli visualised. Some studies compared more than two sputum collection methods, concurrently providing data for different research questions. Discrepancies were resolved by discussion among the authors.

### Data analysis

Spot, pooled, and early morning sputum collections were assessed. Spot sputum collection refers to rapid, on-demand sputum collection during a single consultation, regardless of the time of day, usually at a health centre. For this study, pooled sputum collection refers to sputum that was pooled from each spontaneous expectoration into the same sputum container over a period of several hours. Early morning sputum collection refers to sputum expectorated in the morning after waking. WHO guidelines recommend that two spot sputum samples are collected during the same day,^18^ and this approach is widely practised because it reduces the risk of patients being lost to follow-up during testing.^19^ By contrast, pooled and early morning sputum collection generally require additional clinic visits: first to provide a sputum container and request the collection, then another visit for the sputum to be received for testing.

There is no gold standard sputum collection method, so the local standard (or control) sputum collection method for tuberculosis diagnosis in the reference group was considered the reference standard. In most studies, the reference group collected spot sputum samples (appendix pp 7–10).

All analyses were done with STATA, version 13. All diagnostic performance data were calculated at the sputum-sample level, except where indicated because the authors reported only the data at the individual patient-level. Positivity for each test was defined by the authors of each study. The diagnostic performance of sputum collection methods was analysed to calculate the odds ratio (OR) of the likelihood of a positive laboratory result, with 95% CI, displayed in forest plots. The threshold for significance was p less than 0·05. All analyses were done separately for each of the laboratory tests used to diagnose tuberculosis (microscopy, culture, and PCR).

Pairwise meta-analyses were done with a random-effects model by use of the DerSimonian and Laird method when there were two or more studies that investigated the same predefined research question with the same laboratory test, because significant heterogeneity was expected.^20^ Heterogeneity was assessed visually by forest plots, and analytically by *I*^2^ and Cochrane Q test.

We did a meta-regression analysis using a logistic regression model of the study effect sizes if there was significant heterogeneity in the pairwise meta-analysis. To evaluate other factors that might have contributed to the likelihood of a positive tuberculosis diagnostic test, another meta-regression was done to evaluate the study or sputum collection characteristics, including components of the instructions given that affected the odds of a positive tuberculosis diagnostic test in the study reference groups. The categories included in both meta-regressions are shown in the appendix (p 12). We imputed missing data for the meta-regression using the average result of the other studies. If national tuberculosis incidence or HIV co-infection rates were not reported by a study, we used published data from WHO or the Centres for Disease Control and Prevention (CDC) from the closest available year for the country where the study took place.^21^, ^22^, ^23^

When pairwise meta-analyses revealed more than one method with significantly different diagnostic performance to the reference group, we did a Bayesian network meta-analysis to identify the method with the best overall diagnostic performance^24^ using the “mvmeta” and “network” STATA commands.^25^ Publication bias was assessed with funnel plots and rank Egger's test.^26^ The methodological quality of the studies was assessed with the QUADAS-2 checklist, because analysing diagnostic performance is similar to studying diagnostic accuracy.^27^

### Role of the funding source

The funders of the study had no role in study design, data collection, data analysis, data interpretation, or writing of the report. The corresponding author had full access to all the data in the study and had final responsibility for the decision to submit for publication.

## Results

**Figure 1:**
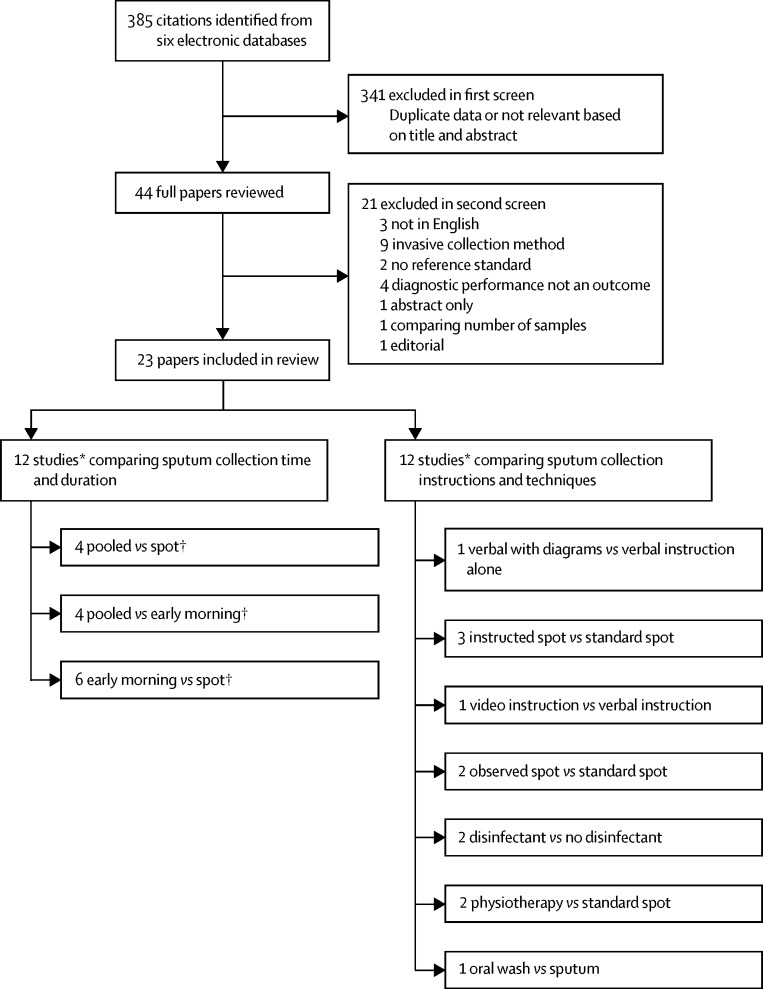
Study selection *Findings from one study contributed to assessment of both aspects of collection methods: sputum collection time and duration; and sputum collection instructions and techniques. †One study compared pooled sputum collection versus spot sputum collection versus early morning sputum collection, and was therefore included in all meta-analyses comparing sputum collection time and duration.

Eligibility criteria were fulfilled by 23 studies published from 1959 to 2017, comprising 8967 participants who provided 19 252 sputum samples (figure 1; table 1). A within-patient design, in which each participant provided sputum for both the intervention and reference groups, was used by 57% (13 of 23) of the studies; 83% (19 of 23) of the studies took place in low-income or middle-income countries (table 1).

**Table 1:** Study characteristics

	**Country**	**Study design**	**Microscopy stain**	**Processing before microscopy**	**Culture method**	**TB diagnosis at recruitment**	**Number of participants**	**Female participants (%)**	**Number of samples**	**Participants recruited during treatment (%)**	**National TB prevalence (per 100 000)**	TB HIV co-infection (%)*
Mpagama et al (2012)^28^	Tanzania	Within patient	ZN	Decontamination and centrifugation	Not done	Suspected	50	28	150	0	609	16†
Andrews and Radhakrishna (1959)^29^	India	Within patient	Fluorescent	None	Solid	Confirmed	348	31	1392	NR	465	0
Majumdar et al (1962)^30^	India	Within patient	ZN	None	Solid	Confirmed	76	NR	222	42	465	0
Warren et al (2000)^31^	USA	Before and after intervention	Fluorescent	Decontamination and centrifugation	Solid	Confirmed	65	NR	221	32	7·9	15
Abdel Aziz et al (1985)^32^	Egypt	Within patient	ZN	None	Not done	Suspected	90	NR	360	NR	82	0·01
Krasnow and Wayne (1969)^33^	USA	Within patient	Not done	Not done	Solid	Suspected	261	NR	1336	NR	19	0
Kestle and Kubica (1967)^34^	USA	Within patient	Not done	Not done	Solid	Suspected	183	NR	366	NR	23	0
Ssengooba et al (2012)^35^	Uganda	Within patient	ZN	NS	Liquid	Suspected	1862	43	3724	NR	170	50
Pande et al (1974)^36^	India	Within patient	ZN	None	Solid	Confirmed	160	38	320	38	465	0
Geldenhuys et al (2014)^37^	South Africa	Within patient	Fluorescent	Decontamination and centrifugation	Liquid	Suspected	600	50	1068	NR	696	22†
Schoch et al (2007)^38^	Switzerland	Within patient	ZN	Centrifugation only	Solid and liquid	Suspected	101	25	167	0	206	5
Khan et al (2007) ^39^‡	Pakistan	Within patient	ZN	NS	Not done	Suspected	1520	NR	2819	0	384	0·40
Khan et al (2007) ^39^‡	Pakistan	RCT	ZN	NS	Not done	Suspected	3055	49	5632	0	384	0·40
Alisjahbana et al (2005)^40^	Indonesia	RCT	ZN	NS	Not done	Suspected	174	45	505	0	775	2·70
Sakundarno et al (2009)^41^	Indonesia	Before and after intervention	NS	NS	Not done	Suspected	508	NR	1168	NR	715	4·60
Mohamed et al (2014)^42^	Sudan	RCT	ZN	NS	Not done	Suspected	328	NR	656	NR	151	3·40
Maciel et al (2009)^43^	Brazil	Case-control	Not done	Not done	Solid and liquid	Not reported	119	45	119	100	58	20
Bell et al (2009)^13^	Malawi	Within patient	ZN	Decontamination and centrifugation	Solid	Suspected smear-negative	111	NR	220	0	368	68†
Souza and Bammann (2007)^44^	Brazil	Within patient	ZN	Decontamination and centrifugation	Solid	Suspected	132	NR	264	12	58	100*
Lee et al (2013)^45^	South Korea	RCT	NS	NS	NS	Suspected	77	NR	228	0	106	0·40
Mhalu et al (2015)^46^	Tanzania	RCT	Fluorescent	NS	Not done	Suspected	200	47	200	NR	528	25
Kalema et al (2012)^47^	Uganda	RCT	ZN	None	Solid	Suspected	220	52	440	0	170	80†
Peres et al (2011)^48^	Brazil	RCT	Not done	Not done	Solid and liquid	Suspected	120	NR	240	NR	57	17
Davis et al (2009)^49^	Uganda	Within patient	PCR	Not done	Not done	Suspected	127	NR	254	0	108	46†

TB=tuberculosis. ZN=Ziehl–Neelsen. Confirmed= microbiological confirmation of tuberculosis diagnosis. NR=not reported. NS=not specified. RCT=randomised control trial.

* National data were used in this variable, unless indicated otherwise.

† Data reported within the study.

‡ This study assessed instructed spot versus uninstructed spot sputum collection, but also carried out a subanalysis comparing instructed early morning versus instructed spot sputum collection.

Diagnostic testing by sputum microscopy was assessed in 18 studies, of which 67% (12 of 18) used Ziehl–Neelsen staining with light microscopy, 22% (four of 18) used fluorescent microscopy, and 11% (two of 18) did not provide this information. Tuberculosis culture was assessed in 14 studies: 57% (eight of 14) used solid culture, 14% (two of 14) used liquid culture, 21% (three of 14) used both, and 7% (one of 14) did not specify the culture method. PCR was assessed in one study.

Sputum collection time and duration were assessed by studies that compared pooled versus spot sputum collection (n=4), pooled versus early morning sputum collection (n=4), and early morning versus spot sputum collection (n=6; figure 1). Findings from one study were included in all three comparisons. Pooled sputum was collected overnight, for 24 h, or for 72 h (appendix p 7).

**Figure 2:**
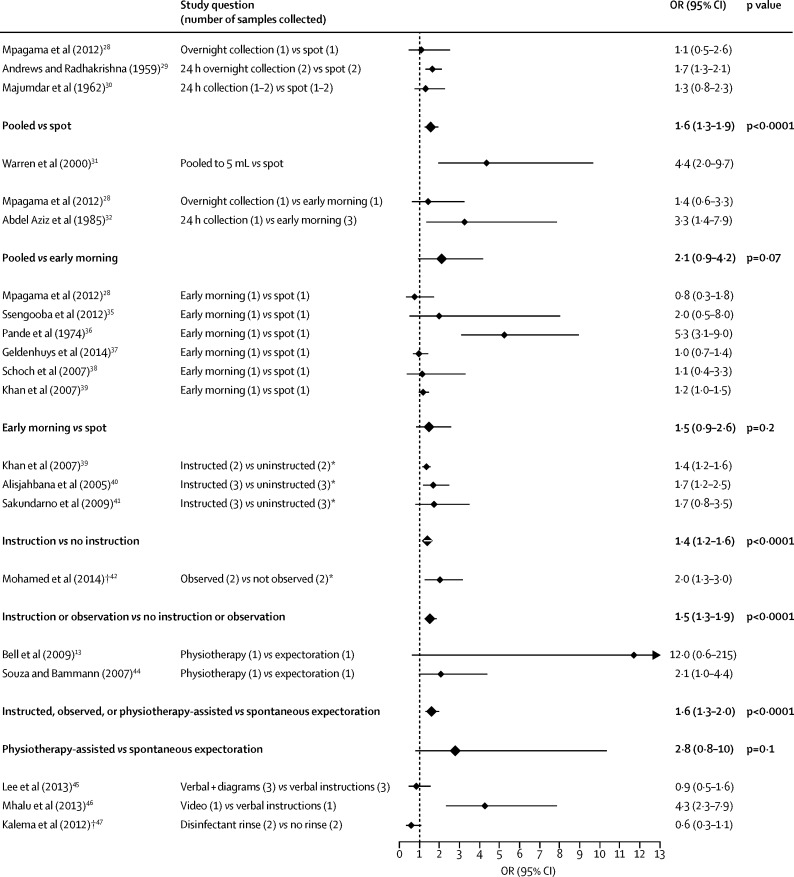
Forest plot of both individual and combined meta-analysis results for smear microscopy *Samples collected in both comparison and reference group were a combination of spot and early morning samples. †Odds ratio (OR) was calculated from individual patient-level data and not from sputum sample-level data. Comparisons in bold typeface and a larger diamond symbol indicate the results of a pairwise meta-analysis.

**Figure 3:**
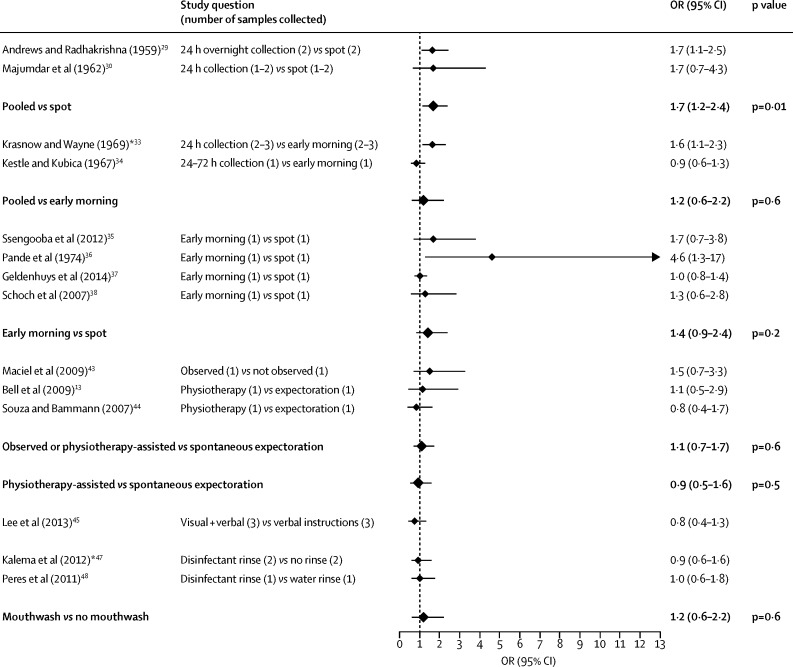
Forest plot of both individual and combined meta-analysis results for mycobacterial culture *Odds ratio (OR) was calculated from individual patient-level data and not from sputum sample-level data. Comparisons in bold typeface and a larger diamond symbol indicate the results of a pairwise meta-analysis.

Figure 2, Figure 3 show that, compared with spot sputum collection, pooled collection significantly increased the odds of a positive result with sputum microscopy (OR 1·6, 95% CI 1·3–1·9, p<0·0001) and culture (1·7, 1·2–2·4, p=0·01). These study results had no significant heterogeneity (p=0·5 for sputum microscopy and p=1·0 for culture; table 2). One study, which compared spot sputum collection versus pooled collection until a volume of 5 mL sputum had been collected, was excluded from both pairwise and subsequent network meta-analyses (and from heterogeneity assessments) because this collection method was substantially different from those of other studies.

**Table 2:** Summary of main findings of pairwise and network meta-analyses

		**Pooled *vs* spot**	**Instructed spot *vs* uninstructed spot**	**Early morning *vs* spot**	**Pooled *vs* early morning**
**Microscopy**					
Pairwise meta-analyses		Three studies	Six studies	Six studies	Two studies
	Number of samples	1764	8445	8248	510
	OR (95% CI)	1·6 (1·3–1·9)	1·6 (1·3–2·0)	1·5 (0·9–2·6)	2·1 (0·9–4·2)
	p value	<0·0001	<0·0001	0·2	0·07
	Heterogeneity *I*^2^, p value	0%, 0·5	25%, 0·3	84%, <0·0001	45%, 0·2
Pairwise meta-analyses*		··	··	Five studies	··
	Numbers of samples	··	··	7928	··
	OR (95% CI)	··	··	1·1 (1·0–1·3)	··
	p value	··	··	0·2	··
	Heterogeneity *I*^2^, p value	··	··	0%, 0·7	··
Network meta-analysis		Three studies	Six studies	Six studies	Two studies
	OR (95% CI)	1·8 (1·1–3·0)†	1·8 (1·2–2·8)	1·4 (0·9–2·1)†	1·3 (0·7–2·4)
	p value	0·03	0·01	0·2	0·4
**Culture**					
Pairwise meta-analyses		Two studies	Three studies	Four studies	Two studies
	Number of samples	1614	603	5279	1702
	OR (95% CI)	1·7 (1·2–2·4)	1·1 (0·1–1·7)	1·4 (0·9–2·4)	1·2 (0·6–2·2)
	p value	0·01	0·6	0·2	0·6
	Heterogeneity *I*^2^, p value	0%, 1·0	0%, 0·6	50%, 0·1	81%, 0·02

OR=odds ratio.

* Excluding outliers.

† Comparison versus uninstructed spot.

Pooled sputum collection tended to have higher odds of a positive microscopy result than did early morning sputum collection (OR 2·1, 95% CI 0·9–4·2, p=0·07; figure 2). This was not the case for culture results (OR 1·2, 95% CI 0·6–2·2, p=0·6; figure 3). However, one of these two studies only reported individual patient-level data.^33^ This pairwise meta-analysis had significant heterogeneity (*I*^2^=81%, p=0·02) but there were only two studies in this analysis, so a meta-regression could not be done.

Studies comparing spot versus early-morning sputum collection had the largest total sample size and showed no significant difference in diagnostic performance for microscopy (8248 samples, OR 1·5, 95% CI 0·9–2·6, p=0·2; figure 2) or culture (5279 samples, 1·4, 0·9–2·4, p=0·2; figure 3). There was significant heterogeneity in the effect size within the studies in the pairwise meta-analysis for sputum microscopy (*I*^2^=84%, p<0·0001), but not for culture (*I*^2^=50%, p=0·1; table 2). Meta-regression (appendix p 11) revealed that this heterogeneity in studies that used microscopy was explained by one study that included only participants with microbiologically confirmed tuberculosis (coefficient 1·5, 95% CI 0·7–2·3, p=0·006), whereas all other studies included participants with suspected tuberculosis. Exclusion of this study did not affect the pattern of significance (OR 1·1, 95% CI 0·95–1·3, p=0·7, *I*^2^=0%; table 2).

Sputum collection instructions and techniques were assessed by reviewing studies that compared instruction given before sputum collection versus no instruction (n=3); instruction given during observation by a health-care professional versus no instruction (n=2); instruction provided while a physiotherapist assisted versus unassisted expectoration (n=2); verbal instructions with diagrams versus only verbal instructions (n=1); video versus verbal instructions (n=1); disinfectant mouthwash before sputum collection versus no disinfectant mouthwash (n=2); and use of mouthwash versus sputum as a diagnostic sample (n=1; figure 1).

Instructions given for sputum collection (for either intervention or reference groups) varied greatly between all studies and are shown in the appendix (p 7–10): 35% (eight of 23) of the studies did not mention the instructions, if any, given to the reference group. The advice given in the intervention groups to produce sputum included spontaneous expectoration, taking numerous deep breaths and coughing, holding breath for a second and coughing, drinking a hot drink before collection, or physiotherapy-assisted pressure on the chest accompanied by slow expiration. One study found that, compared with verbal instructions, there was no difference in the odds of positive microscopy if the instructions were given with diagrams (OR 0·9, 95% CI 0·5–1·6, p=0·6), whereas another study found video instructions to be associated with improved odds (4·3, 2·3–7·9, p<0·0001; figure 2).

Pairwise meta-analyses showed that instructions on how to produce sputum or emphasising the difference between sputum and saliva (verbally or visually) increased the odds of a positive microscopy result (OR 1·4, 95% CI 1·2–1·6, p<0·0001, figure 2), with no significant heterogeneity (p=0·4). None of these studies used culture for diagnosis. There were not enough studies to investigate whether observing patients while collecting sputum affected the diagnostic performance of microscopy or culture. In addition, the only study^42^ that used microscopy was analysed with individual patient-level data.

Compared with standard spot sputum collection, physiotherapy-assisted collection did not significantly affect diagnostic performance with microscopy or culture (Figure 2, Figure 3), although these studies were small (243 participants in total). When these interventions were considered as differing levels of guidance to assist participants to produce spot or early morning sputum, then the odds of a positive microscopy result increased cumulatively: 1·4 times (95% CI 1·2–1·6, p<0·0001) by instructions; 1·5 times (1·3–1·9, p<0·0001) by instructions or observing the participant; and 1·6 times (1·3–2·0, p<0·0001) by a combination of instruction, observation, and physiotherapy (figure 2), with no significant heterogeneity (p=0·2). Not enough studies have been published for a meaningful equivalent analysis for mycobacterial culture (figure 3).

Mouth washing with disinfectant was evaluated by two studies, and this intervention did not demonstrably affect the odds of a positive result with microscopy or culture (Figure 2, Figure 3). One of these studies was analysed with individual patient-level data.^47^

The study that assessed PCR^49^ showed that, although tuberculosis could be diagnosed by testing oral rinse samples, this technique had lower odds of a positive PCR result than did spot sputum collection (OR 0·56, 95% CI 0·34–0·93). This study was not included in any meta-analysis or following meta-regression, because it used a different laboratory test and collection method.

Meta-regression of factors predicting diagnostic performance in the reference group was possible because all studies used either spot collection, early morning collection, or a combination of both as their reference group (appendix pp 7–10). This analysis showed that early morning sputum collection did not affect the variation in the odds of a positive microscopy (p=0·6) or culture test in the reference group (p=0·7; appendix p 12). The only factor that increased the odds of a positive microscopy (p=0·04) or culture test (p=0·004) in the reference group was if the study recruited only individuals with microbiologically confirmed tuberculosis. Increasing prevalence of HIV co-infection, and studies done in recent years decreased the odds of a culture result. More recent studies were also associated with decreased odds of a positive microscopy test (appendix p 12).

**Figure 4:**
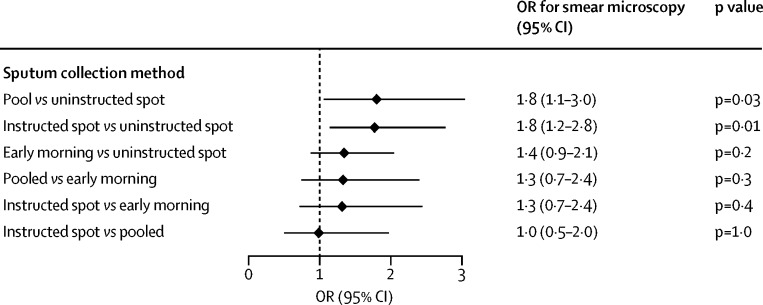
Forest plot of results of the network meta-analysis OR=odds ratio.

Network meta-analysis was possible only for the results of sputum microscopy in 14 studies, by use of their 17 comparative assessments as shown in table 2. These studies assessed uninstructed spot, pooled, instructed (including observed and physiotherapy assisted) spot, and early morning sputum collection. Four studies assessing the value of instructions, shown in figure 2, requested a combination of spot and early morning samples in both comparison and reference groups (appendix pp 7–10). However, the pairwise meta-analysis comparing early morning collection with spot sputum collection showed no significant difference in diagnostic performance (table 2). Thus, these studies in the network meta-analysis were considered as comparisons of uninstructed versus instructed spot samples. The network meta-analysis showed that, compared with uninstructed spot collection, the odds of positive sputum microscopy were increased by pooled sputum samples (OR 1·8, 95% CI 1·1–3·0, p=0·03) or by instructed spot sputum collection (1·8, 1·2–2·8, p=0·01; figure 4). The network meta-analysis estimated that there was no difference in the odds of a positive sputum microscopy between pooled and instructed spot collection (OR 1·0, 95% CI 0·5–2·0, p=1·0; figure 4). Pooled (47%) and instructed (46%) spot collections had similar likelihood of being the best method of obtaining a positive microscopy test within the network meta-analysis.

**Figure 5:**
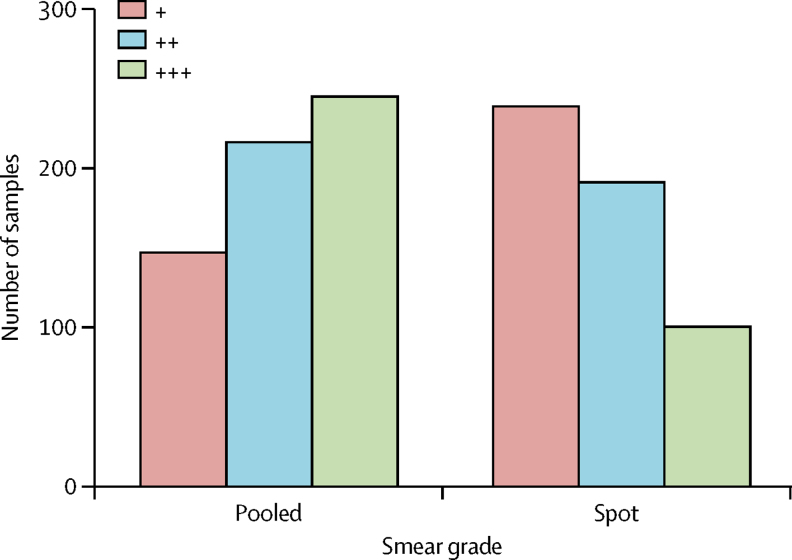
Comparison of the differences in mycobacterial load within smear microscopy positive samples between pooled and spot sputum collections This analysis involved combining the results reported by Andrews and Radhakrishna (1959)^29^ and Majumdar and colleagues (1962),^30^ which showed 75% (608 of 807) of positive smear microscopy results in pooled sputum collections compared with 66% (530 of 807) in spot sputum collections. Smear grade is indicated by the number of + symbols. Because only aggregate data for these studies were published, matched analysis was not possible. Therefore, the p value (<0·0001) was calculated from a Wilcoxon-Mann-Whitney rank sum test.

Meta-analysis of the grade^9^ of positivity of diagnostic test results was possible only for the effect of pooled sputum collection on sputum microscopy, because this was the only research question addressed by at least two studies. Combining results of these two studies showed that pooled sputum collection significantly increased both the positivity and the grade of positivity of sputum microscopy compared with uninstructed spot sputum collection (figure 5).

Funnel tests did not show any asymmetry, and no small-study effects were observed with Egger's test for microscopy (p=0·4) or culture (p=0·7; appendix p 13).

Potential bias was identified for 65% (15 of 23) of studies (table 3). The main sources of bias were selection of participants from different target populations and fixed order of collection techniques, which could have introduced systematic error.

**Table 3:** Study quality assessed by the risk of bias with the QUADAS-2 checklist

	Selection of patients*	Interpretation of comparison sputum collection method*	Interpretation of control sputum collection method*	Patient flow*
Mpagama et al (2012)^28^	Low	Low	Low	High
Andrews and Radhakrishna (1959)^29^	High	Low	Low	Low
Majumdar et al (1962)^30^	High	Unclear	Unclear	Unclear
Warren et al (2000)^31^	High	High	Low	High
Abdel Aziz et al (1985)^32^	Unclear	High	Low	High
Krasnow and Wayne (1969)^33^	Low	Low	Low	Unclear
Kestle and Kubica (1967)^34^	Unclear	Low	Low	Low
Ssengooba et al (2012)^35^	High	Unclear	Unclear	Low
Pande et al (1974)^36^	Low	Low	Low	High
Geldenhuys et al (2014)^37^	High	Low	Low	High
Schoch et al (2007)^38^	Low	Low	Low	High
Khan et al (2007)^39^	Low	Low	Low	Low
Alisjahbana et al (2005)^40^	Low	Unclear	Unclear	Low
Sakundarno et al (2009)^41^	High	High	High	High
Mohamed et al (2014)^42^	Low	High	High	Low
Maciel et al (2009)^43^	High	Low	Low	High
Bell et al (2009)^13^	Low	High	Low	High
Souza and Bammann (2007)^44^	Low	Low	Low	High
Lee et al (2013)^45^	Low	Low	Low	Low
Mhalu et al (2015)^46^	Low	High	Unclear	Low
Kalema et al (2012)^47^	Low	Low	Low	Low
Peres et al (2011)^48^	Low	Low	Low	Low
Davis et al (2009)^49^	Low	Low	Low	Low

* Stages at which bias could have been introduced.

## Discussion

This systematic review and meta-analysis showed that, compared with standard spot sputum collection, providing instructions or collecting a pooled sputum sample increased the odds of tuberculosis diagnosis in microscopy and culture by 1·6–1·8 times. It is unsurprising that diagnosis of pulmonary tuberculosis depends on the quality of the sputum sample tested. The effect of these simple, inexpensive strategies on diagnostic performance was similar to that of the relatively expensive GeneXpert MTB/RIF test, which, in the largest published studies, increased the odds of diagnosing tuberculosis by 1·3–1·5 times^50^, ^51^, ^52^ (appendix p 14). Although molecular tests are useful in detecting drug resistance and in reducing the variability of interpretation by laboratory technicians, there seems to be more interest in developing and implementing new commercial tests than in strengthening existing methods. For example, a search of the PubMed database for studies published in the past 5 years suggests that, for tuberculosis diagnosis, 79 studies have focused on the effect of the GeneXpert MTB/RIF test, whereas only seven have focused on non-invasive sputum collection techniques (appendix p 15). Although these techniques relate to different aspects of the diagnostic pathway, and PubMed-indexed publications do not represent all tuberculosis-related publications, these comparisons suggest that increased attention and resources should be invested into studying how sputum is collected, alongside the development of new diagnostic products.^53^

In 2007, the case definition of smear positivity changed to include cases with the presence of at least one acid-fast bacillus in at least one sputum sample.^54^ Although most studies did not define the threshold of positivity used in smear microscopy, we assumed that six studies done after 2007 would be affected by this change. The studies varied in the number of samples collected from each participant, so positivity in this analysis was, where possible, defined for each sample and not for individual cases, and ORs were calculated instead of sensitivity and specificity. Furthermore, our meta-regression analyses showed that this change in the definition of smear positivity did not contribute to the heterogeneity within the study effect sizes, or the variability in the likelihood of a positive test demonstrated between the reference groups. A positive smear does not equate to a diagnosis of tuberculosis, as false-positive results arise because acid-fastness is not unique to *M tuberculosis*. Therefore, culture, which is unaffected by this change in definition for smear-positive microscopy, is the gold standard for confirming tuberculosis and showed similar results to microscopy in our meta-analyses.

Two sputum samples are sufficient for tuberculosis testing, because approximately 97% of microscopy-positive tuberculosis cases are identified by the first two samples provided.^7^ A meta-analysis^11^ concluded that these two samples could be collected during the same day with no decrease in diagnoses. Globally, 16% (95% CI 13–18) of individuals suspected of having tuberculosis are estimated to be lost to follow-up before starting treatment, so ideally a diagnostic specimen should be collected from patients on the same day as their first assessment.^19^ Providing instruction before or during a spot sputum collection considerably increased tuberculosis diagnosis. Specifically, the evidence from our study shows that explaining (verbally or visually) the difference between saliva and sputum, providing guidance on how to produce sputum, advising breathing exercises, or providing physiotherapy assistance almost doubled the odds of diagnosing tuberculosis. Although the instructions in these studies included common elements, as shown in the appendix (pp 7–10), further research is needed to ascertain which instructions optimally increase diagnostic performance.

Unfortunately, same-day, single-visit tuberculosis diagnosis is not always possible because sputum might not be produced, because of perceptions of superior early morning collection, or because of health system constraints delaying testing and dissemination of results.^55^ Therefore, people being tested for suspected tuberculosis are usually asked to return for follow-up appointments before diagnosis is confirmed. Under these circumstances, our analysis shows that tuberculosis diagnosis could be increased if patients are asked to pool sputum spontaneously expectorated over several hours. Requiring another visit does have costs, risks loss to follow-up, and might not increase the number of people on treatment because many patients start treatment empirically.^53^, ^55^ Yet we can presume that enabling laboratory confirmation of tuberculosis, especially with drug susceptibility results, is likely to be cost-effective because of the avoidance of inappropriate therapy, associated catastrophic costs, and generation of drug resistance, although this needs to be specifically studied.^53^, ^56^, ^57^ Pooled sputum collection increased not only the proportion of positive results but also how strongly positive the microscopy results were, most probably by increasing the likelihood of collecting sputum from the lower respiratory tract, compared with the contents of the upper airways and saliva. We have commenced research to test whether the increased tuberculosis diagnoses associated with pooled sputum over several hours are additive to the advantages provided by instructing spot sputum collection. This is particularly important because comparisons between pooled and spot sputum samples were most influenced by one study^29^ that requested sputum or saliva, which could have influenced the effect size. Pooling sputum collection need not increase the risk of nosocomial tuberculosis transmission if patients are advised to complete sputum collection in well ventilated open-air spaces, away from health-care workers and other people.^58^, ^59^

Patients being tested for tuberculosis are often asked to complete early morning sputum collection, consistent with some international recommendations.^10^ Early morning sputum collections have been thought to be superior to standard spot sputum collection, perhaps because of the diurnal variation in sputum volume and symptoms in other inflammatory lung diseases, together with the assumption that poor lung clearance while sleeping leads to increased expectoration of sputum upon waking.^60^, ^61^, ^62^ However, our analyses show that early morning sputum collection had no significant effect on diagnostic performance, in keeping with previous findings that two spot sputum samples had equivalent diagnostic accuracy to spot plus early morning sputum samples.^11^ Thus, to improve diagnostic performance, instructed spot or pooled sputum samples can be collected at any time.

Strengths of this study include the broad inclusion criteria (reducing selection bias and capturing more research), the absence of major small-study bias (as indicated by the funnel-plot analysis), pairwise meta-analysis of subgroups with a random-effects model (to control for heterogeneity), and the large sample size (most studies were adequately powered and the meta-analysis included >19 000 samples). Limitations include the paucity of good-quality studies, especially those involving culture or PCR; the fact that some studies assessed combined interventions and so the cumulative effect on diagnostic performance could not be measured; and concerns about the robustness of network meta-analysis.^24^, ^63^ However, these limitations were offset by the broadly consistent findings of the pairwise meta-analyses and network meta-analysis, and by inclusion of research assessing both microscopy and culture positivity. To assess the true effect of these collection methods in clinical practice, routine programmatic evaluations are required.

The key implications of this systematic review and pairwise and network meta-analyses are that non-invasive sputum collection methods involving instruction or pooled sputum collection considerably increase tuberculosis diagnosis and that sputum can be collected at any time, not necessarily in the early morning. These inexpensive, simple interventions warrant further research and policy emphasis because their effects are potentially similar to those of comparatively expensive interventions such as investing in new diagnostic products.
