# Correction to *Lancet Glob Health* 2017; 5: e760–71

**DOI:** 10.1016/S2214-109X(17)30255-3

**Published:** 2017-07-14

*Datta S, Shah L, Gilman RH, Evans CA. Comparison of sputum collection methods for tuberculosis diagnosis: a systematic review and pairwise and network meta-analysis.* Lancet Glob Health *2017; **5:** e760–71*—In this Article, the wrong version of the appendix was included. The appendix has been corrected as of July 14, 2017.

