# Supplementary material for: Comparison of sputum collection methods for tuberculosis diagnosis: a systematic review and pairwise and network meta-analysis
Source: Lancet Glob Health. 2017 Jun 15;5(8):e760–71. doi: 10.1016/S2214-109X(17)30201-2 (PMC5567202; doi:10.1016/S2214-109X(17)30201-2)
Supplement: Supplementary appendix [file mmc1.pdf]

THE LANCET
Global Health

Supplementary appendix

This appendix formed part of the original submission and has been peer reviewed. We post it as supplied by the authors.

Supplement to: Datta S, Shah L, Gilman RH, Evans CA. Comparison of sputum collection methods for tuberculosis diagnosis: a systematic review and pairwise and network meta-analysis. *Lancet Glob Health* 2017; published online June 15. http://dx.doi.org/10.1016/S2214-109X(17)30201-2.

ONLINE SUPPLEMENT 1. Full description of the instructions provided to participants in each arm of the studies included in this review. Note ml=millilitre

	Comparison group			Reference group		
First author, year	Sample type	Number of samples collected	Instructions given	Sample type	Number of samples collected	Instructions given
Mpagama, 2012[45]	Pool (Overnight)	1	"Collect in a wide mouth container"	Spot or Early morning	1	"Collect in a wide mouth container"
Andrews, 1959[38]	Pool (Overnight /24 hour)	2	"Asked to fill half of the container with sputa or all saliva if scanty"	Spot	2	"Produced either sputum or saliva on demand within a minute or 2 between 07:30 - 13:00"
Majumdar, 1962[46]	Pool (24 hour)	1 or 2	"Aided by health care worker"	Spot	1 or 2	"Asked to produce sputum"
Warren, 2000[47]	Pool (5 ml)	Unknown	"Accepted samples with more than 5ml of sputum. If less than 5 ml, sample kept for 48 hours and when another sample arrived pooled them to 5 ml"	Spot	Unknown	Not specified
Abdel-Aziz, 1985[48]	Pool (24 hour)	1	"Collect in a wide mouth container. Advised to collect true sputum from the depth of their chest"	Early morning	3	"Collect in a wide mouth container. Advised to collect true sputum from the depth of their chest"
Krasnow, 1969[28]	Pool (24 hour)	2 or 3	Not specified	Early morning	2 or 3	Not specified
Kestle, 1967[49]	Pool (24-72hour)	1	"Collect sample not exceeding 40-50 ml"	Early morning	1	"Collect any sputum raised after waking not exceeding 10 ml"
Ssengooba, 2012[50]	Early morning	1	Not specified	Spot	1	Not specified
Pande, 1974[51]	Early morning	1	"Produce sample in the early hours within a matter of few minutes before cleaning mouth"	Spot	1	"Collect sputum or saliva in a wide mouth container"
Geldenhuys, 2014[52]	Early morning	1	"Asked to take deep breath in and hold for 1 second then cough deeply and vigorously when breathing out. Advised to collect thick mucoid sputum, not saliva or nasal secretions"	Spot	1	"Asked to take deep breath in and hold for 1 second then cough deeply and vigorously when breathing out. Advised to collect thick mucoid sputum, not saliva or nasal secretions"

Schoch, 2007[53]	Early morning	1	"One hour was allowed for spontaneous expectoration of sputum"	Spot	1	"One hour was allowed for the production of spontaneous sputum"
Khan, 2007[54]	Spot and Early morning	2	"Take 3 deep breaths, then cough deeply, aim for 5 ml. Emphasised the importance of sputum rather than saliva providing visual difference"	Spot and Early morning	2	No instructions given
Alisjahbana, 2005[55]	Spot and Early morning	3	"Explained the importance of sputum examination and instructed patients on how to produce adequate samples. A picture of good quality sputum (purulent and thick) was shown and they were specifically instructed to produce at least 5 ml of sputum"	Spot and Early morning	3	"Give 3 samples to the laboratory spot, morning, and spot"
Sakundarno, 2009[56]	Spot and Early morning	3	"Healthcare workers were trained about advising patients to collect samples greater 3 ml of the colour and consistency of sputum"	Spot and Early morning	3	"Healthcare workers were not trained"
Mohamed, 2014[29]	Spot and Early morning	2	"Advised to rinse the mouth, clear the nose and throat, try a hot drink, place both hands in the waist. Take 2-3 deep breaths and cough. Participants were observed"	Spot and Early morning	2	Not specified
Maciel, 2009[57]	Early morning	1	"Advised to use a toothbrush to remove food residue prior to collecting 10 ml. Participants were observed"	Early morning	1	"Advised to collect first morning sample after fasting the previous night"
Bell, 2009[13]	Physiotherapy assisted spot	1	"Deep breaths were taken whilst the physiotherapist carried out cupped clapping on chest, shake for 5 minutes and then asked to blow with pursed lips"	Spot	1	"Collect self-expectorated sputum sample under direct observation"

Souza, 2007[58]	Physiotherapy assisted spot	1	"This manoeuvre is based on slow expiration from the functional residual capacity with the glottis open and the patient in lateral decubitus. The physiotherapist stands behind the patient and uses the right hand to exert counter-supportive pressure on the superolateral rib cage. With the open palm of the left hand, the physiotherapist exerts counter-supportive pressure on the opposite lateral-costal wall. This technique was performed 12 times for approximately 20 minutes per session."	Spot	1	Not specified
Lee, 2013[63]	Early morning	3	"After rinsing the mouth collect expectorated sputum from the lungs after productive cough in morning." Instructions given verbally with diagrams.	Early morning	3	"After rinsing the mouth collect expectorated sputum from the lungs after productive cough in morning." Instructions given verbally.
Mhalu, 2015[60]	Early morning	1	4 min video in Swahili explaining that the collection required secretions from bronchial tree, and not saliva, demonstrating both. Advised to inhale and exhale deeply 3 times and cough after the last breath. This should be done on waking after inhaling steam. Also demonstrates the volume.	Early morning	1	"Sit or stand in an open space, inhale deeply 2-3 times, breathe hard each time and cough as hard as possible after the last breath."

Kalema, 2012[30]	Spot	2	"Prior to sputum expectoration, patients either performed a 60 second oral rinse with chlorhexidine followed in immediate succession by another 60 second rinse with nystatin. Participants were observed"	Spot	2	"Emphasised the importance of sputum rather than saliva providing visual difference. Advised to take 3 deep breaths, then cough deeply, aim for 5 ml. Participants were observed. For the early morning samples advised to do it on awakening. "
Peres, 2011[61]	Early morning	1	"Advised to rinse their mouths with 10 ml of a commercially available mouthwash solution for 1 minute before sample collection"	Early morning	1	"Advised to washed hands with soap and water and rinsed their mouths with tap water before collecting the specimens"
Davis, 2009[62]	Spot	1	"For the collection of oral wash specimens, subjects were instructed to cough vigorously 5 times, then gargle 10 ml of sterile saline for 60 seconds"	Spot	1	Not specified

ONLINE SUPPLEMENT 2. Data extraction form

Please see uploaded Supplement 2.

ONLINE SUPPLEMENT 3. Meta-regression assessing factors that contributed to the heterogeneity in effect size of studies that used sputum smear microscopy to compare early morning versus spot collections of sputum. The meta-analysis of these 6 studies had a chi^2 32 p<0.01, and I-squared 84%. Statistical significance (P<0.05) is indicated by bold font.

	Coefficient	95% confidence interval	P value	Residual I^2 heterogeneity
TB prevalence (per 100,000 people)	-0.0009	-0.0059 - 0.0041	0.6	87%
TB/HIV coinfection (%)	-0.0088	-0.067 - 0.50	0.7	86%
Female participants (%)	0.01	-0.10 - 0.12	0.8	87%
Recruited only microbiologically-confirmed TB cases	**1.5**	**0.7 - 2.3**	**0.006**	**0%**
Studies carried out after the change in “Smear positive” definition	-0.63	-2.3 – 1.0	0.4	85%
Participants recruited during treatment (%)	**0.04**	**0.02 - 0.06**	**0.006**	**0%**
Fluorescent microscopy used	-0.51	-2.8 - 1.8	0.6	86%
Instructions given to study either or both arms	-0.53	-2.3 - 1.2	0.4	78%

ONLINE SUPPLEMENT 4. Meta-regression assessing factors that contributed to the variation demonstrated in the likelihood of a positive diagnostic test within the reference groups of each study. Not applicable results are represented by NA. Statistical significance or notable trends (P<0.08) are indicated by bold font

	Smear microscopy (n=20)			Culture (n=14)		
	Coefficient	95% confidence interval	P value	Coefficient	95% confidence interval	P value
National TB prevalence (100,000 people)	0.00020	-0.0038 - 0.0042	0.9	0.0023	-0.0035 - 0.0081	0.4
TB HIV co-infection (%)	-0.033	-0.072 - 0.0066	0.1	**-0.031**	**-0.063 - 0.0006**	**0.05**
Year	**-0.051**	**-0.10 – 0.003**	**0.04**	**-0.068**	**-0.012 - -0.03**	**0.002**
Studies carried out after the change in “Smear positive” definition	-0.31	-2.3 – 1.7	0.7	NA	NA	NA
Female participants (%)	-0.069	-0.20 - 0.060	0.3	-0.057	-0.24 - 0.13	0.5
Recruited only microbiologically-confirmed TB cases	**2.2**	**-0.088 - 4.2**	**0.04**	**3.5**	**1.4 - 5.6**	**0.004**
Participants recruited during treatment (%)	0.048	-0.018 - 0.11	0.1	0.017	-0.027 - 0.061	0.4
Fluorescent microscopy used	1.0	-1.3 - 3.3	0.4	NA	NA	NA
Sample concentration prior to microscopy	-0.51	-2.1 – 2.0	1.0	NA	NA	NA
Liquid culture used	NA	NA	NA	**-2.3**	**-4.4 – 0.12**	**0.04**
Breathing manoeuvre to aid sputum production	-0.11	-2.8 – 2.5	0.9	-0.32	-3.8 - 3.2	0.8
Instructed on sputum quality	-0.21	-2.2 - 1.8	0.8	-0.88	-3.5 - 1.8	0.5
Early-morning samples collected	0.45	-1.5 - 2.4	0.6	0.42	- 2.2 - 3.0	0.7
Number of samples collected per patient	0.53	-0.66 - 1.7	0.4	0.90	-0.92 - 2.7	0.3

FOOTNOTE. The meta-regression analysed the odds of positive smear microscopy or culture in the reference group. It included the variables shown above and: research question, type of instructions, and sputum processing methods. The effect of liquid culture on the odds of a positive culture result lost its association when the year of the study was included in the meta-regression (Coefficient=-0.82, 95% confidence interval=-2.9, 1.2 and p=0.4). Note that n indicates the number of analyses versus a reference standard that were included in this meta-regression, which is different from the number of publications (see Figure 1).

ONLINE SUPPLEMENT 5. Funnel plots assessing publication bias in studies investigating the effects of sputum collection methods on the diagnostic yield of sputum smear microscopy (top) and culture (bottom).

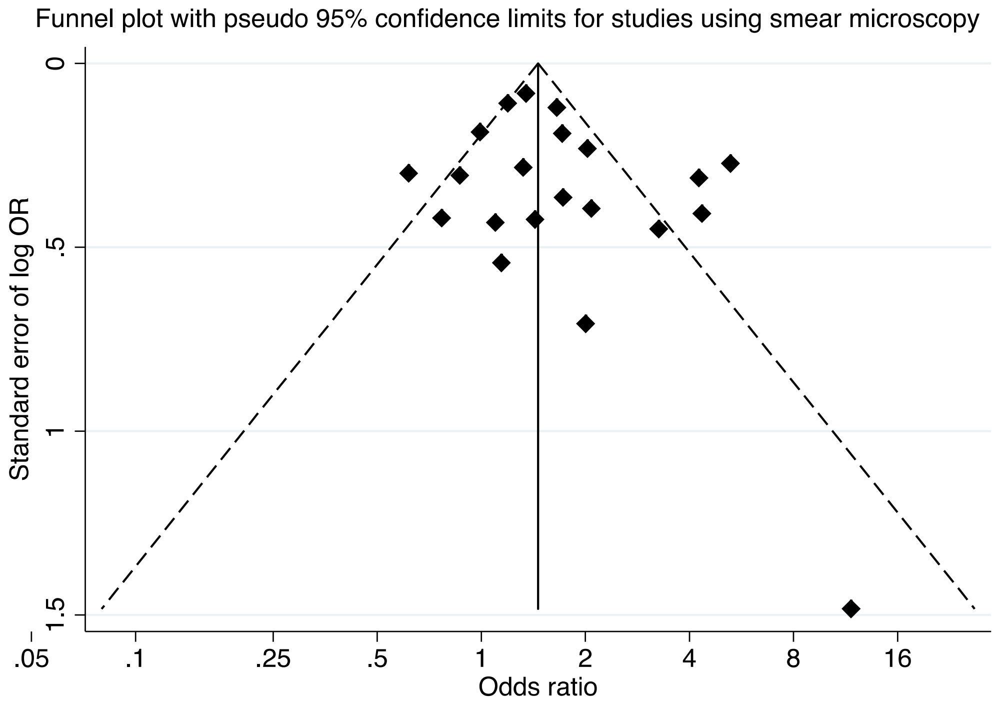

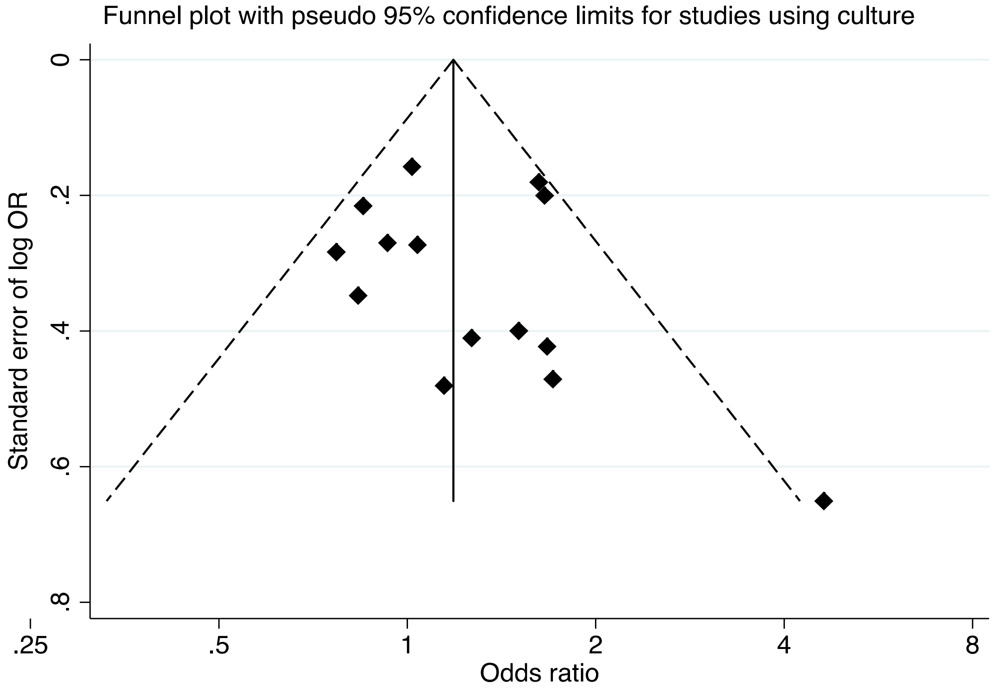

ONLINE SUPPLEMENT 6. A Table demonstrating the results from the largest, international studies comparing GeneXpert versus sputum smear microscopy, and a meta-analysis that calculated the incremental yield of GeneXpert compared to sputum smear microscopy.

First author, year	Study site	Study type	Participant	Number of participants	Percentage GeneXpert positive (n)	Percentage smear microscopy positive (n)	Odds ratio (95% CI)
Boehme, 2010[31]	International multi-site	within patient	Adults suspected of pulmonary TB	1730	39% (675)	33% (567)	1.3 (1.1 - 1.5)
Boehme, 2011[32]	International multi-site	within patient	Adults suspected of pulmonary TB	6069	15% (933)	12% (699)	1.4 (1.3 - 1.6)
Steingart, 2014[33]	Hypothetical 2.5% prevalence	meta-analysis	Adults suspected of pulmonary TB	1000	2% (22)	2% (16)	1.4 (0.72 - 2.7)
Steingart, 2014[33]	Hypothetical 5.0% prevalence	meta-analysis	Adults suspected of pulmonary TB	1000	4% (44)	3% (33)	1.4 (0.85 - 2.1)
Steingart, 2014[33]	Hypothetical 10% prevalence	meta-analysis	Adults suspected of pulmonary TB	1000	9% (88)	7% (65)	1.4 (1.0 - 1.9)
Steingart, 2014[33]	Hypothetical 30% prevalence	meta-analysis	Adults suspected of pulmonary TB	1000	26% (264)	20% (195)	1.5 (1.2 - 1.8)

ONLINE SUPPLEMENT 7. Table demonstrating the number of peer-reviewed publications involving the impact of non-invasive sputum collection methods or GeneXpert on pulmonary TB diagosis.

Theme associated with the diagnosis of pulmonary TB	PubMed search terms	Number of PubMed results in last 5 years	Number of publications relevant to theme
Non-invasive sputum collection methods	((pulmonary tuberculosis[Title/Abstract] AND diagnosis[Title/Abstract]) AND sputum[Title/Abstract]) AND (clearance[Title/Abstract] OR collection[Title/Abstract] OR submission[Title/Abstract] OR acquisition[Title/Abstract]))	23	7
GeneXpert	(pulmonary tuberculosis[Title/Abstract] AND diagnosis[Title/Abstract]) AND (Xpert[Title/Abstract] OR GeneXpert[Title/Abstract])	100	79
